# Hypochlorite-induced porcine model of peritoneal fibrosis through the activation of IL1β-CX3CL1-TGFβ1 signal axis

**DOI:** 10.1038/s41598-020-68495-0

**Published:** 2020-07-13

**Authors:** Yu-Ting Hsu, Ching-Ho Wu, Chun-Yuan Chao, Yu-Syuan Wei, Yen-Chen Chang, Yi-Ting Chen, Shuei-Liong Lin, Su-Yi Tsai, Ya-Jane Lee, Pei-Shiue Tsai

**Affiliations:** 1 Department of Veterinary Medicine, School of Veterinary Medicine, National Taiwan University, Taipei, 10617 Taiwan, ROC; 2 Graduate Institute of Veterinary Medicine, School of Veterinary Medicine, National Taiwan University, Taipei, 10617 Taiwan, ROC; 3 Graduate Institute of Veterinary Clinical Science, School of Veterinary Medicine, National Taiwan University, Taipei, 10617 Taiwan, ROC; 4 Graduate Institute of Molecular and Comparative Pathobiology, School of Veterinary Medicine, National Taiwan University, Taipei, 10617 Taiwan, ROC; 5 Department of Internal Medicine, National Taiwan University Hospital, Taipei, 10002 Taiwan, ROC; 6 Department of Integrated Diagnostics & Therapeutics, National Taiwan University Hospital, Taipei, 10002 Taiwan, ROC; 7 Graduate Institute of Physiology, College of Medicine, National Taiwan University, Taipei, 10051 Taiwan, ROC; 8 Department of Life Science, College of Life Science, National Taiwan University, Taipei, 10617 Taiwan, ROC; 9 Research Center for Developmental Biology and Regenerative Medicine, National Taiwan University, Taipei, 10617 Taiwan, ROC

**Keywords:** Kidney, Kidney diseases, Animal physiology, Chronic kidney disease, Continuous renal replacement therapy, Peritoneal dialysis

## Abstract

Patients with kidney failure rely on life-saving peritoneal dialysis to facilitate waste exchange and maintain homeostasis of physical conditions. However, peritoneal dialysis often results in peritoneal fibrosis and organ adhesion that subsequently compromise the efficiency of peritoneal dialysis and normal functions of visceral organs. Despite rodent models provide clues on the pathogenesis of peritoneal fibrosis, no current large animal model which shares high degree of physiological and anatomical similarities to human is available, limiting their applications on the evaluation of pre-clinical therapeutic efficacy. Here we established for the first time, hypochlorite-induced porcine model of peritoneal fibrosis in 5-week-old piglets. We showed that administration 15–30 mM hypochlorite, a dose- and time-dependent severity of peritoneal fibrosis characterized by mesothelium fragmentation, αSMA^+^ myofibroblasts accumulation, organ surface thickening and type I collagen deposition were observed. We also demonstrated in vitro using human mesothelial cells that hypochlorite-induced fibrosis was likely due to necrosis, but not programmed apoptosis; besides, overexpression of IL1β, CX3CL1 and TGFβ on the peritoneal mesothelium in current model was detected, similar to observations from peritoneal dialysis-induced peritoneal fibrosis in human patients and earlier reported mouse model. Moreover, our novel antemortem evaluation using laparoscopy provided instant feedback on the progression of organ fibrosis/adhesion which allows immediate adjustments on treatment protocols and strategies in alive individuals that can not and never be performed in other animal models.

## Introduction

Patients with kidney dysfunction rely on the peritoneal membrane to perform life-saving peritoneal dialysis (PD); structural and functional integrity of the peritoneum determine the efficiency of PD; however, dialysis itself often triggers inflammatory and fibrosis processes that progressively results in PD failure^1–3^. Scar or fibrosis could occur in both parietal and visceral peritoneum, and in some rare cases, may result in life threatening encapsulating peritoneal sclerosis (EPS), a catastrophic complication with obscure pathogenesis and high mortality rate if competing risk of death is not taken into account^1,2^. In other cases, intra-abdominal procedures can lead to peritoneal fibrosis (PF) and adhesions between peritoneal contents that subsequently disturb intestinal functions^4^. The common pathogenesis of PF following PD or intra-abdominal procedures is characterized by the loss of mesothelium, infiltration of increasing numbers of macrophages and α-smooth muscle actin (αSMA)-positive myofibroblasts, and thickening of the sub-mesothelial (SM) region with collagen accumulation^5–9^. Despite earlier studies suggested the occurrence of epithelial mesenchymal transition (EMT) in the fibrosis process of peritoneum^10,11^, recent study using lineage tracing technique demonstrated that the major source of peritoneal myofibroblasts is the resident SM fibroblasts rather than mesothelial cells undergoing EMT as suggested in some studies^6^. Nevertheless, after acute mesothelium injury, the mesothelium can be partially repaired by remnant surviving mesothelial cells, which give rise to promising expectation for regenerative cell therapy^6^.

Development of effective treatments and therapies requires a basic understanding of disease conditions. In most cases, diseases or specific physical conditions can be replicated in animal models of non-human species. In terms of organ size, anatomical and physiological similarities; porcine is a better animal for modeling human diseases when compare with murine species; moreover, the immune system of pigs is similar to that of human and can yield better understanding on the pathogenesis of the diseases^12,13^. Therefore, pigs have been widely used as animal models for studying cardiovascular disease^14^, hepatic cirrhosis^15^, wound repair^16^, diabetes^17^, ophthalmology^18^, respiratory medicine^19^, and kidney transplantation^20^.

Although pig models are useful for translational medicine, no porcine model has been established for PF, uremia and/or therapeutic evaluations. It is known that chlorhexidine gluconate or methylglyoxal can be used to induce PF in rodent; however, these above-mentioned approaches require repeated and/or constant exposures of stimuli^21,22^. In this study, we aim to establish peritoneal fibrosis in pig by a single administration of sodium hypochlorite (NaClO), and establish evaluation methods including antemortem laparoscopy examination and biopsy, postmortem necropsy, pathological and cytokine evaluations to assess mesothelium integrity, collagen deposition, thickening of SM compact zone and myofibroblast accumulations. With this novel porcine model and detail investigation on the mechanism of hypochlorite-induced peritoneal fibrosis, we can facilitate and validate existing efficacy evaluations on potential target compounds and therapeutic protocols, and provide an alternative pre-clinical evaluation platform for validating findings from murine models as well as accessing feasibility, efficiency and clinical safety of regenerative cell therapy prior to human trials.

## Results

### Antemortem laparoscopic examination revealed fibrotic organ surface and tissue adhesions in NaClO-injured pigs

**Figure 1:**
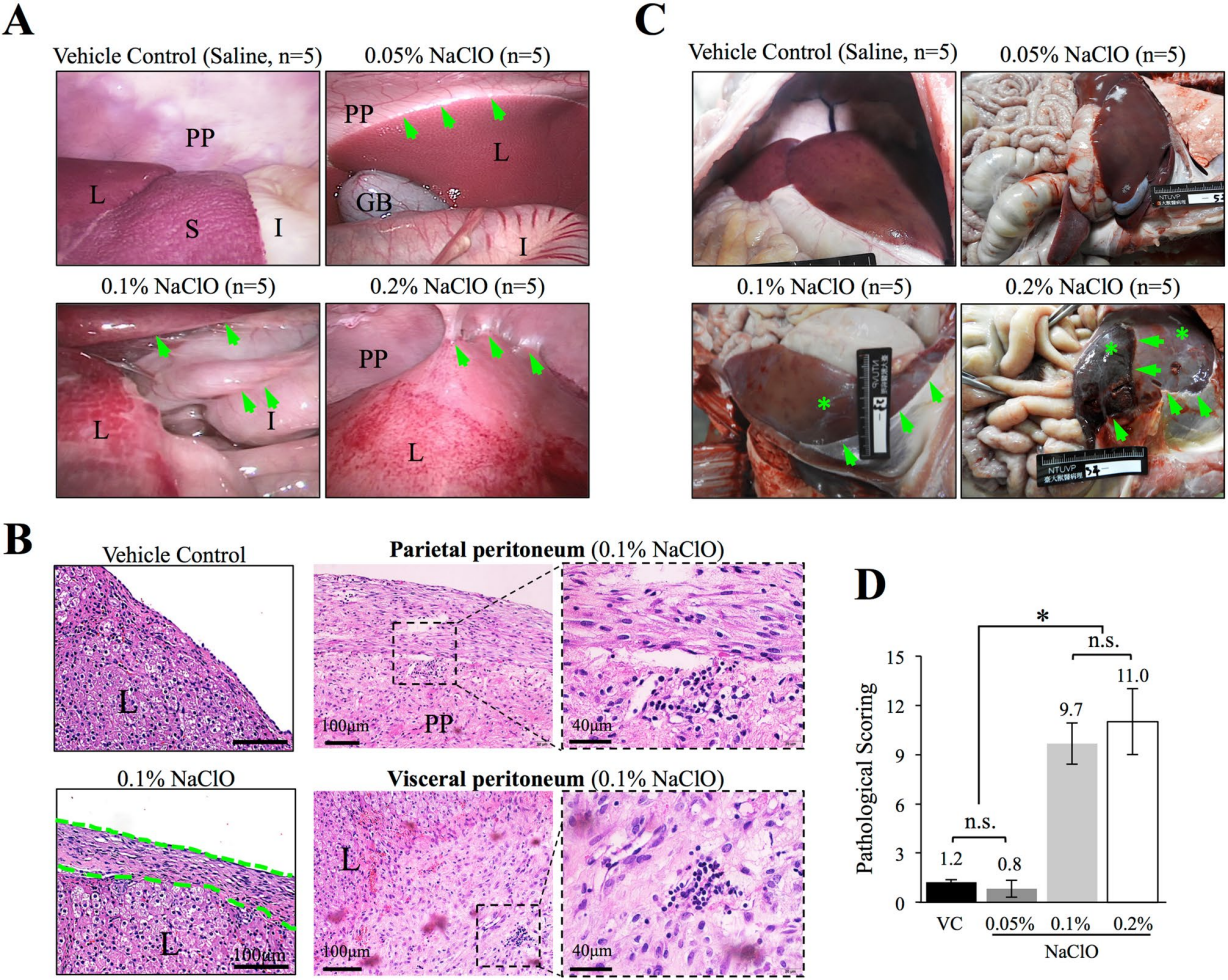
Ante-mortem laparoscopy and post-mortem necropsy evaluations of NaClO-induced peritoneal fibrosis. (**A**) Laparoscopy evaluation of abdominal cavity showed multi-point adhesions (indicated with arrows) between parietal peritoneum and fibrotic visceral organs. (**B**) Infiltration of spindle cells with increased thickness at the surface of the liver was observed in liver sample obtained from 0.1% NaClO-injured pigs. Parietal and visceral peritoneum biopsies obtained upon laparoscopic evaluation were processed for H&E stain. Accumulation and thickening of fibrous connective tissue with lymphocytes infiltration were observed in pigs injured with 0.1 NaClO, representative images from 0.1% NaClO-injured pig were illustrated. (**C**) Gross findings of abdominal organs in pigs treated with different concentrations of NaClO. Saline was used as vehicle control. Arrows indicated adhesions between visceral organs or between parietal peritoneum with organs. Fibrosis on the surface of the organs was also observed and marked with asterisks. (**D**) Quantitative pathological evaluations by modified adhesion scoring system showed the lesions in 0.1 or 0.2% NaClO-injured pigs were statistically more severe than saline- or 0.05% NaClO-injured pigs. N.S.: non-significant different. Asterisk indicates significant difference (p < 0.05) between groups. *L* liver; *I* intestine; *GB* gall bladder; *S* spleen; *PP* parietal peritoneum. Representative images from 5 individual pigs of each group were presented.

Pre-injection ultrasonography (Fujifilm SonoSite, USA) was first carried out to assess the (ab)normality of peritoneal cavity, and was also used to monitor peritoneal injection procedure of NaClO. An apparent anechoic area (marked with asterisk) was observed between liver and intestine indicated successful administration of NaClO in the abdominal cavity (Fig. S1A). Antemortem evaluation of peritoneal cavity was carried out by laparoscopic examinations 7 days post NaClO injections and prior to euthanasia. While no apparent gross lesions were observed in control (saline) and 0.05% NaClO-injured pigs (Fig. 1A, supplementary videos 1–2), dose-dependent increase on the severity of gross lesion, such as organ surface fibrinous deposition, multifocal adhesions (marked with arrows) between liver capsule, parietal peritoneum, and serosa of intestine, were observed in pigs injured with 0.1 and 0.2% NaClO (Fig. 1A, supplementary videos 3–4). Liver and peritoneum biopsies obtained upon laparoscopic evaluation showed a normal histology in control pig; however, accumulation and thickening of fibrous connective tissue with lymphocytes infiltration were observed in pigs injured with 0.1 and 0.2% NaClO (Fig. 1B).

### Postmortem examination showed dose-dependent increase on pathological changes after NaClO injury

To provide end-point postmortem analyses, pigs were euthanized one-week after the NaClO administration. Grossly, similar to saline-treated control pigs, no apparent gross lesion was noted in 0.05% NaClO-injured pigs (Fig. 1C). However, thickening of hepatic and splenic capsules, fusion of hepatic lobes, multifocal peritoneal adhesions (marked with arrows) between liver, spleen, intestine and parietal peritoneum were noted in 0.1% and 0.2% NaClO-injured pigs (Fig. 1C). Moreover, when pigs injured with 0.1 and 0.2% NaClO, considerable amount of white fibrinous depositions was observed at the surface of visceral organs (Fig. 1C, marked with asterisks). Based on gross pathological scoring system described in Table 1, pathological scoring in 0.1 and 0.2% NaClO-injured pigs were 9.7 ± 1.3 and 11.0 ± 2.0, respectively, and were statistically more severe than that of saline-treated (1.2 ± 0.1) and 0.05% (0.8 ± 0.8) NaClO-injured pigs (Fig. 1D).

**Table 1:** Gross pathological scoring system.

Organ	Description	Score
Liver	Normal	0
	Dull edge	1
	Surface turbid thickening	
	Local	2
	Diffuse	3
	Fusion of lobes	4
Spleen	Normal	0
	Irregular surface	1
	Surface turbid thickening	
	Local	2
	Diffuse	3
Intestine	Normal	0
	Surface turbid thickening	1
	Adhesion	
	Mild	2
	Severe (multiple loops)	3
Adhesions of visceral organs	No adhesion	0
	Peritoneum adhesion with liver	2
	Peritoneum adhesion with spleen	2
	Peritoneum adhesion with intestine	2
	Liver adhesion with spleen	2
	Liver adhesion with intestine	2

### Dose-dependent thickening of both parietal and visceral peritoneum in NaClO-injured pigs

**Figure 2:**
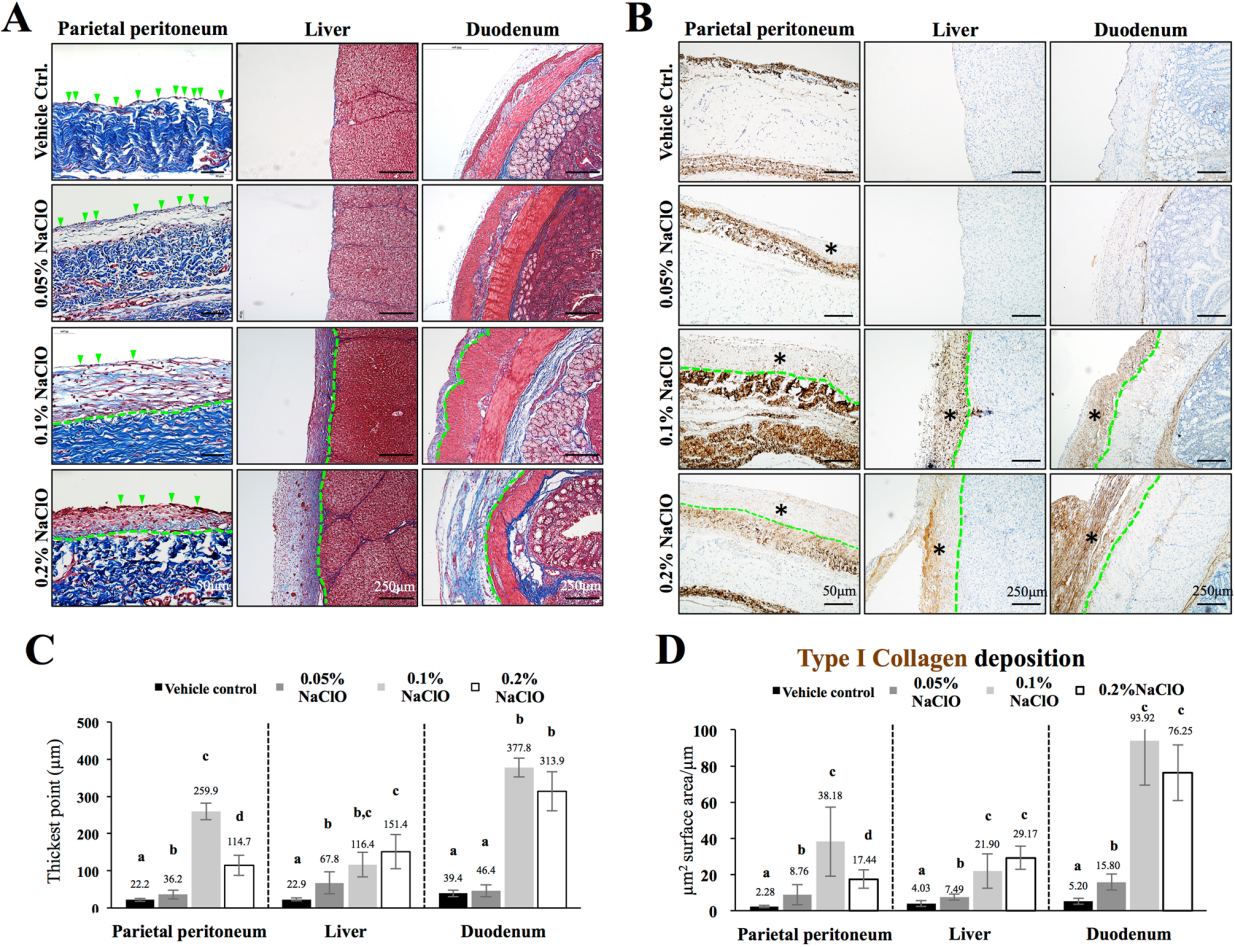
Histological evaluations on the thickening of tissue surface and type I collagen accumulation in NaClO-injured pigs. (**A**) A single lining of surface mesothelial cells (indicated with green arrow heads) was observed above the submesothelial connective tissue layer in both parietal and visceral peritoneum of control pigs. However, a dose-dependent thickening of submesothelial compact zone was observed in NaClO-injured pigs. Distance between the surface mesothelial cell layer (green arrowheads) and submesothelial connective tissue layer (underneath the green dash line) was marked. A dose-dependent thickening of surface capsule was also detected in the liver and duodenum. (**B**) Immunohistochemistry showed a dose-dependent increased type I collagen deposition (marked with asterisks) on the parietal peritoneum and on the surface of visceral organs. (**C**) NaClO-induced organ surface thickening was quantified by CellSens Software and 0.1% NaClO induced the most server pathological changes among all concentration tested. (**D**) In the peritoneum overlying parietal peritoneum and duodenum, 0.1% NaClO resulted in the most severe type I collagen accumulation, and a dose-dependent accumulation of type I collagen was observed on the surface of liver. A–d indicate significant difference (p < 0.05) between groups. Representative images from five individual pigs of each group were presented.

To validate whether postmortem fibrotic appearance and thickening of peritoneum were due to collagen deposition, Masson’s trichrome and type I collagen stain were carried out. As showed in Fig. 2A,B, little to no collagen deposition was observed at the parietal peritoneum (the single layer of the mesothelial cell was marked with arrow heads), liver, and duodenum (visceral peritoneum) in control and 0.05% NaClO-injured pigs. In a sharp contrast, a dose-dependent thickening of collagen-rich fibrotic tissues and type I collagen deposition were observed in 0.1% and 0.2% NaClO-injured pigs (Fig. 2A,B). Moreover, an increase number of spindle-shape cells were also observed in thickened peritoneum of 0.1% and 0.2% NaClO-injured pigs suggested the presence of myofibroblasts in these tissues. Interestingly, unlike in liver and duodenum showed a dose-dependent surface thickening upon NaClO stimulation, a compact stack of spindle cells was accumulated above the layer of SM connective tissue in 0.2% NaClO-injured pigs (Fig. 2A). To further quantify tissue thickening and collagen deposition in NaClO-injured pigs, an objective measurement was performed as described in methods section. As showed in Fig. 2C,D, a dose-dependent peritoneal thickening and type I collagen deposition were noted on the liver and duodenum. However, 0.1% NaClO resulted in the most severe organ surface thickening and type I collagen deposition in the peritoneum overlying ventral parietal peritoneum (Fig. 2C,D).

### Loss of mesothelial cells and accumulation of α-smooth muscle actin positive myofibroblasts in NaClO-injured pigs

**Figure 3:**
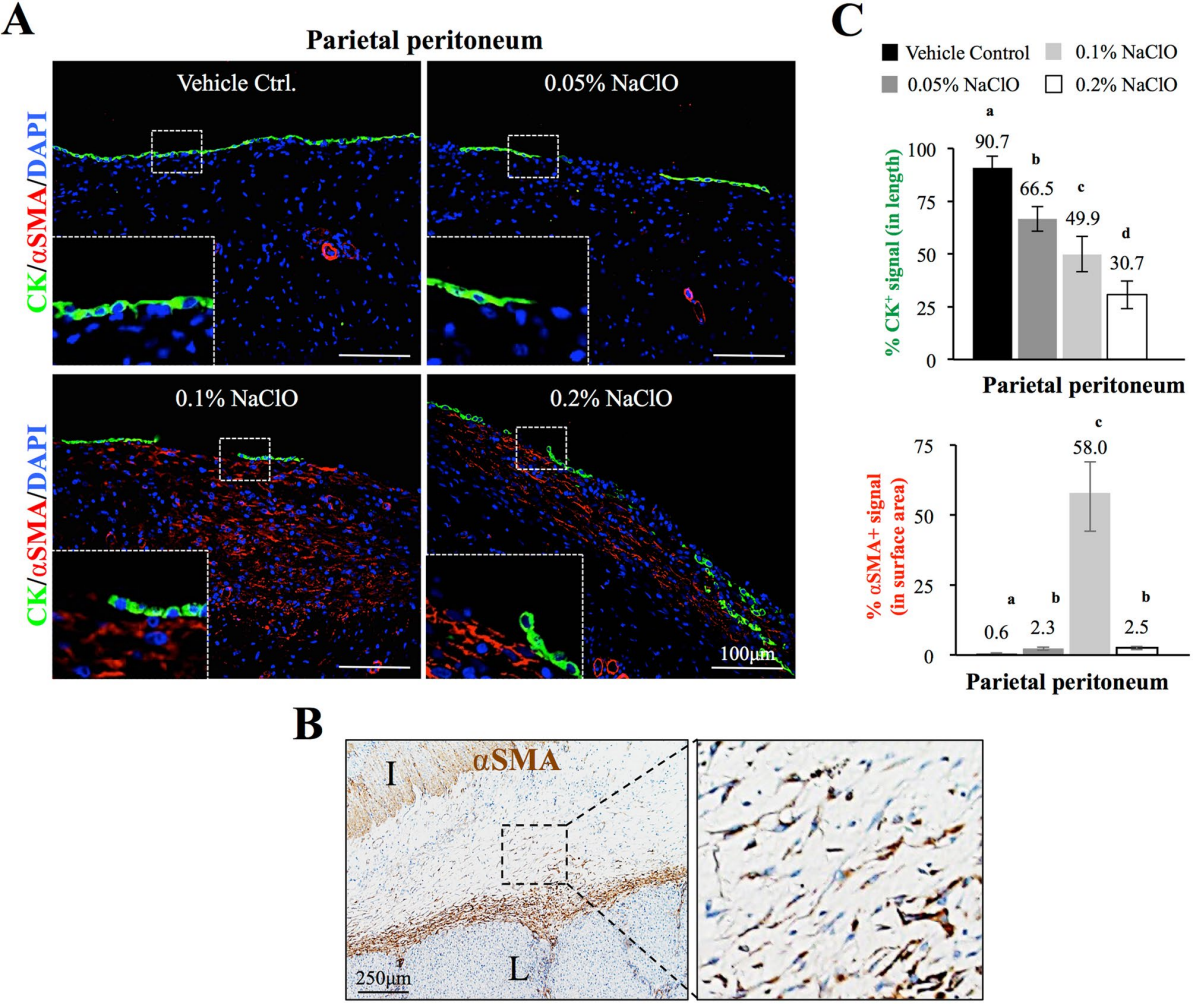
Indirect immunofluorescent evaluation of mesothelium integrity and accumulation of α smooth muscle actin positive (αSMA^+^) cells in NaClO-injured pigs. (**A**) Sodium hypochlorite-induced fragmentation of mesothelium of ventral parietal peritoneum (labeled with cytokeratin in green) and infiltration of αSMA^+^ cells (labeled in red) at the submesothelial region of ventral parietal peritoneum. Higher magnification showed continuation of CK^+^ mesothelial cells with minimal amount of αSMA^+^ cells in control pig, and discontinued mesothelium with αSMA^+^ cells in NaClO-injured groups. (**B**) Immunohistochemistry study demonstratedαSMA^+^ spindle cells infiltrated in the adhesion area between two visceral organs. (**C**) Quantification analyses showed a dose-dependent loss of CK^+^ mesothelium at the surface of parietal peritoneum and liver after NaClO injury. Accumulation of αSMA^+^ cells was more apparent in 0.1% when compared with control or other NaClO-injured pigs. *I* intestine, *L* liver. Representative images from 5 individual pigs of each group were presented. a–d indicate significant difference (p < 0.05) between groups.

To investigate whether NaClO-induced peritoneal injury share similar pathogenesis of PD-induced PF^5–9^, immunofluorescence staining of mesothelial cell marker, cytokeratin (in green) and myofibroblast marker, αSMA (in red) were carried out. As showed in Fig. 3A, a continuous mesothelium labeled with CK was observed throughout the entire surface of parietal peritoneum in control pigs without apparent αSMA^+^ myofibroblast at the SM region; however, fragmentation of mesothelium as well as increase accumulation of αSMA^+^ myofibroblasts were observed after NaClO injury. Magnified images depicted the integrity of mesothelium and the increased infiltration of αSMA^+^ myofibroblasts in parietal peritoneum after NaClO injury (Fig. 3A). Infiltration of αSMA^+^ myofibroblasts was observed not only at the surface of parietal peritoneum, but also in the adhesion between visceral organs (Fig. 3B). As showed in Fig. 3B, these αSMA^+^ myofibroblasts were embedded in collagen-rich tissues indicating these myofibroblasts could be collagen-producing cells as described in our earlier mouse model^6^. Three-dimensional reconstruction images further confirmed the integrity of peritoneal membrane in control pigs without αSMA^+^ myofibroblast infiltration in the SM tissue. Loss of CK^+^ mesothelial cells with an infiltration of αSMA^+^ myofibroblasts underneath the remaining mesothelium was apparent in NaClO-injured pigs (Fig. S1B). Quantitative analyses showed that when compared with control pigs displayed a relatively higher mesothelial coverage rate (90.7 ± 5.5%), pigs injured with 0.05%-0.2% NaClO exhibited a dose-dependent lost of parietal mesothelium (Fig. 3C, 66.5 ± 11.8%, 49.9 ± 8.3% and 30.7 ± 6.6% for 0.05%, 0.1% and 0.2% NaClO, respectively). Furthermore, when the number of αSMA^+^ myofibroblasts was quantified, a significant increase of αSMA^+^ myofibroblasts was detected in 0.1% NaClO-injured pigs (58.0 ± 13.7%) but not in control, 0.05% or 0.2% NaClO-injured pigs (Fig. 3C).

### Hypochlorite led to necrosis of mesothelial cells and systemic inflammatory responses

**Figure 4:**
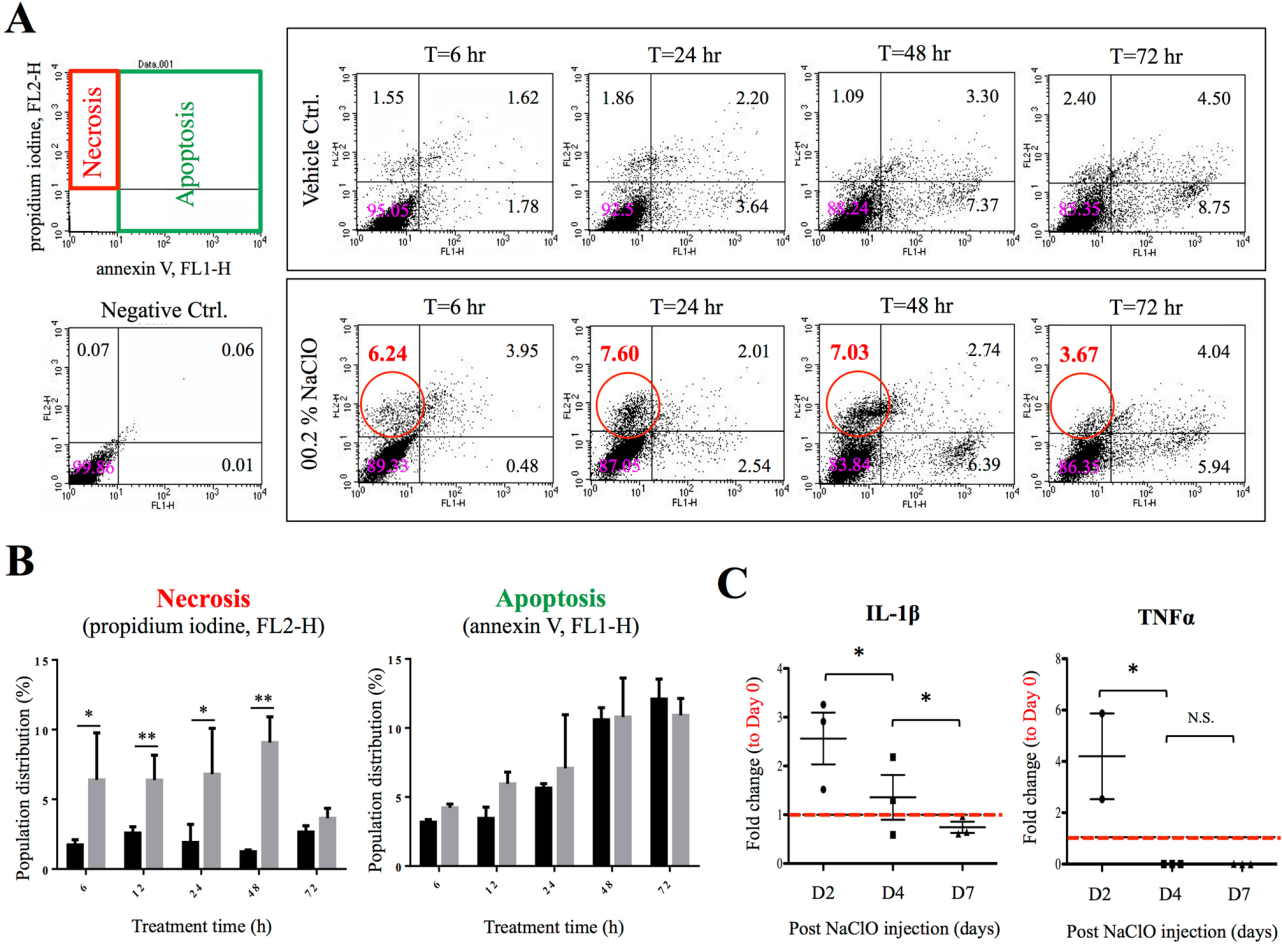
Flow cytometry and cytokine analyses on hypochlorite-induced cell fate. **(A)** Human mesothelial cells were treated with 0.02% NaClO for 6–72 h. Flow cytometry analyses for cellular apoptosis (using annexin V as marker) and necrosis (using PI as marker) showed NaClO mainly induced cell necrosis rather than a programmed cell death. (**B**) Quantitative analysis showed not statistical difference between control and NaClO-treated mesothelial cells; however, significant differences were measured on necrosis between two experimental conditions. (**C**) Cytokine analyses on serum samples obtained from 0.1% NaClO-injured pigs indicated significant elevation of pro-inflammatory cytokines IL-1β and TNF-α at 2–4 days post injury. Asterisks indicate significant differences between groups (*p < 0.05, **p < 0.01, *N.S.* not statistical different).

To investigate cellular mechanism of hypochlorite-induced loss of parietal mesothelium and the deteriorating effect of hypochlorite on cell fate, human mesothelial cell line MeT-5A was used for in vitro experiments, cellular necrosis and apoptosis marker proteins annexin V and propidium iodine (PI) were used for subsequent flow cytometry analysis. As showed in Fig. 4, although a time-dependent increase of apoptosis marker protein annexin V was detected in NaClO-treated cells (from 0.48% at 6 h to 5.94% at 72 h), theses changes did not differ between control and NaClO-treated mesothelial cells at the same time point of evaluation. In contrast, significant increase of necrosis marker, PI was detected when cells were treated with NaClO suggesting NaClO treatment mainly led to necrosis, but not apoptosis of mesothelial cells (Fig. 4A, B). Necrosis is known to initiate inflammatory responses that characterized with the release of inflammatory cytokines from injured cells. As showed in Fig. 4C, when time course experiments were performed and blood samples from 0.1% NaClO-injured pigs were examined for systemic cytokines production, significant elevation of pro-inflammatory cytokines interleukin 1β (IL-1β) and tumor necrosis factor α (TNFα) was detected after 2 days post NaClO administration supported the occurrence of necrosis after NaClO injury (Fig. 4C).

### Time-dependent increase of chemokine CX3CL1 expression on the mesothelium after NaClO injury

**Figure 5:**
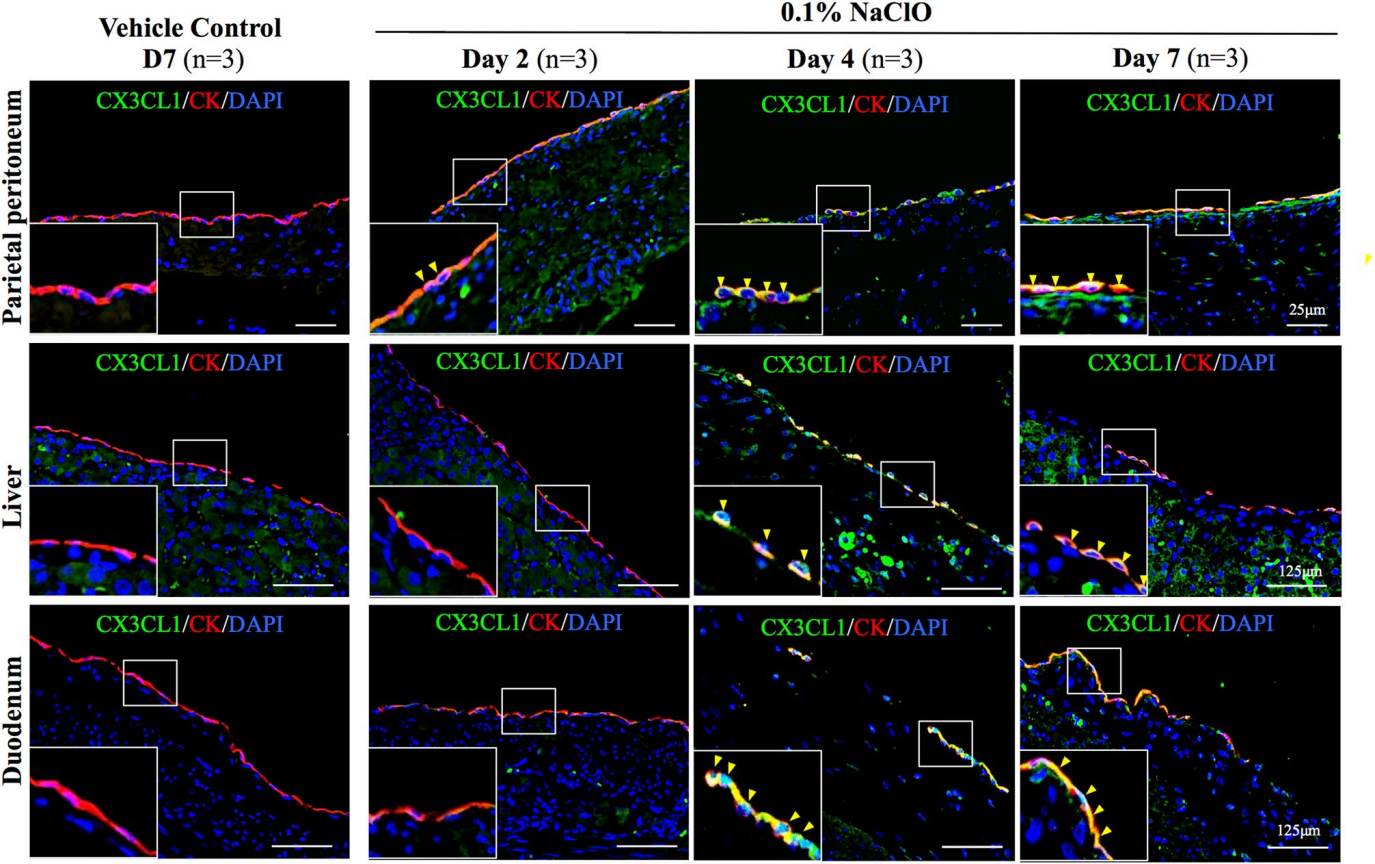
Evaluation of cell surface expression of CX3CL1 expression in NaClO-injured pigs. A time-dependent increase on cell surface fractalkine CX3CL1 protein expression was detected on the mesothelium layer of parietal peritoneum, liver and duodenum. Enlarged image depicted specific CX3CL1 protein expression (in green) on the mesothelial cells (indicated by CK staining in red). Arrowheads indicated co-localization of two staining. For each experimental condition, 10 image frames from 3 different pigs were evaluated (30 images per experimental condition) and representative images were presented.

Recent publication suggested fractalkine receptor (CX3CR1)-CX3CL1 interaction mediated macrophage-mesothelium crosstalk and promoted PD-induced peritoneal fibrosis^23^. To study whether NaClO-induced PF shares similar signal transduction and pathogenesis as those of PD-induced PF, we evaluated mesothelium expression of cell surface CX3CL1 from 0.1% NaClO-injured pigs. As showed in Fig. 5, no CX3CL1 protein expression was detectable in control pigs, and 2 days after NaClO administration, minor CX3CL1 protein expression was observed in parietal peritoneum, liver and duodenum; moreover, an apparent and pronounced CX3CL1 protein expression from both parietal and visceral peritoneum was detected at 4 days and 7 days NaClO-injured pigs (Fig. 5). Similar to earlier report in mouse, we observed an increased co-localization (indicated with arrowheads in magnified images) of cytokeratin (marker for mesothelium) and CX3CL1 from 0.1% NaClO-injured pigs demonstrated NaClO-induced overexpression of fractalkine CX3CL1 in both parietal and visceral mesothelium indicated that NaClO-induced PF and PD-induced PF may at least in part, share similar signal transduction pathway.

### TGF-β1 protein expression was increased in the peritoneum fluid and on the thicken tissue surface in hypochlorite-injured pigs

**Figure 6:**
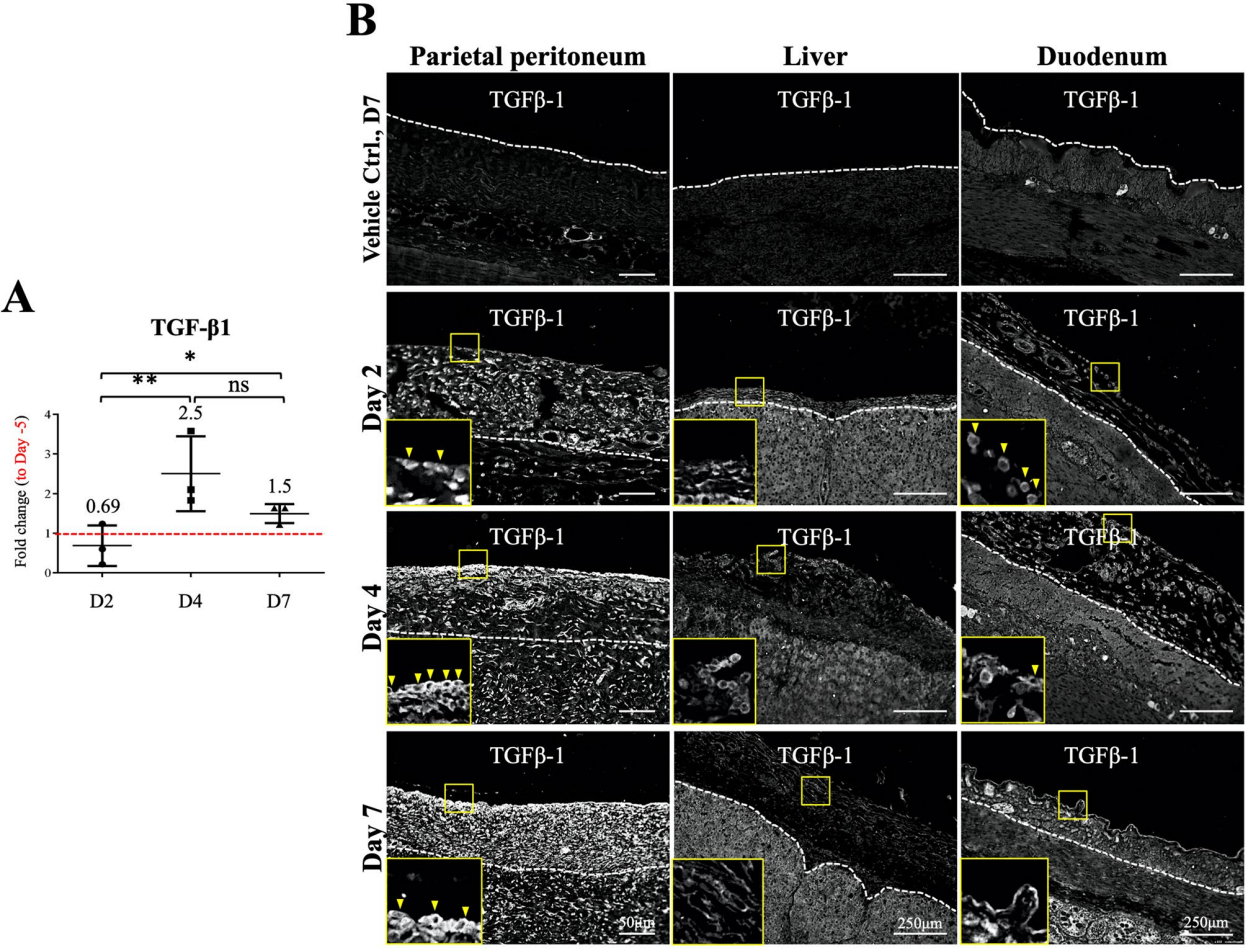
Evaluation of TGF-β1 protein expression in the abdominal lavage and tissues. (**A**) Abdominal lavage was used to evaluate the fibrotic environment of abdominal cavity after NaClO injury. With ELISA, a significant increase in TGF-β1 was detected at day 4 and 7 after NaClO injury. (**B**) Paraffin-embedded tissue sections were used to examined surface and cellular expression of TGF-β1. In line with data from abdominal lavage and Western-blotting analysis, a significant increase in TGF-β1 on parietal peritoneum and duodenum, but not in liver was detected at tissue surface after NaClO injury, no changes on liver was likely due to strong signal present at the parenchyma. Enlarge images showed overexpression of TGF-β1 on the mesothelial cells (indicated with arrowheads). For each experimental condition, 10 image frames from 3 different pigs were evaluated (30 images per experimental condition) and representative images were presented. Asterisks indicate significant differences between groups (*p < 0.05, **p < 0.01, ***p < 0.001).

Activation of TGF-β1 gene and protein expression is one of the main characteristics for organ fibrosis. We showed in Fig. 6A that when abdominal lavage fluid was obtained and examined for the presence of TGF-β1, a significant increase of TGF-β1 was detected by ELISA after 4 days post NaClO-injury (2.5-fold and 1.5-fold increase for day 4 and day 7, respectively) indicated abdominal environment was prone to fibrosis (Fig. 6A). In line with data from abdominal lavage fluid, the increased TGF-β1 protein expression was also observed by indirect immunofluorescent study showing a time-dependent increase on surface TGF-β1 protein expression (Fig. 6B). It is worth noted that although we detected in general, up-regulation of TGF-β1 protein expression in abdominal lavage, tissue staining and tissue homogenates (data not shown) after NaClO-injury, we observed from immunofluorescent study, TGF-β1 was highly expressed in the mesothelium (Fig. 6B, insets, indicated with arrowheads) suggesting the major source of TGF-β1 might be secreted from the remnant injured mesothelium.

## Discussion

Peritoneal fibrosis resulted from PD or peritoneal injury hampers the efficiency of PD and alters normal function of visceral organs. Despite mechanisms and pathogenesis of peritoneal fibrosis have been studied; most of the information was derived from experiments using rodent species^24–26^. For patients who undergo PD and develop simple sclerosis or in a rare situation, EPS, innovated, effective and safe therapies to prevent or to cure PF are urgently needed. In this study, we established for the first time, porcine model of PF and provided alternative evaluation methods for not only validating findings from murine models and but also providing a potential large animal model for future evaluation on the feasibility, efficacy and clinical safety of therapeutic options. When compared with mouse model showed 0.05% NaClO (7.5 mM) led to apparent PF^6^, we showed in pigs, pronounced fibrosis and significant surface thickening of parietal peritoneum or visceral organs occurred only at the higher concentrations of 0.1–0.2% (15–30 mM) NaClO. Moreover, unlike in rodent models, adhesions between intestines or liver lobes were observed only in pigs injured with higher NaClO concentrations of 0.1% and 0.2%, but not at 0.05% NaClO^6,27^. These discrepancies likely due to differences on the nature immunological responses to stimuli between pig and rodent species as we observed infiltration of neutrophils and elevation of pro-inflammatory cytokines IL-1β and TNF-α only when > 0.1% NaClO were applied; therefore, pigs which share similar immune system to that of human may share similar pathogenesis of diseases in human patients^12,13^. Nevertheless, in agreement with observations in mouse, we demonstrated in our pig model that a dose-dependent increase of surface type I collagen accumulation, fragmentation and loss of mesothelial cells and infiltration of αSMA^+^ myofibroblasts at the SM region in NaClO-injured pigs. Our data demonstrated that unlike other fibrosis models require repeated stimuli in a relatively long period of time^28^, our PF model can be established in pig within a week by a single administration of NaClO.

Earlier study used lineage tracing technique demonstrated that the main source of peritoneal myofibroblasts is SM resident fibroblasts instead of mesothelial cells transformation to fibroblast via EMT process^6^. In agreement with that, we showed the presence and the infiltration of αSMA^+^ myofibroblasts at the SM layer and in the adhered junctions of visceral organs without overlapping with signals from CK^+^ cells supporting that EMT was not occurred in current model. From immunofluorescent study, we also showed that at 0.1% NaClO, the presence and the accumulation of αSMA signals at the SM compact zone were most apparent among dosages tested. The discrepancies between necropsy and histological findings in 0.2% NaClO group with less accumulation of αSMA at the SM compact zone in compared with 0.1% NaClO were likely due to higher concentration of NaClO led to acute loss of mesothelium without the recruitment of αSMA^+^ myofibroblasts as described in chronic fibrotic process^6^.

Recent study by Helmke et al. showed CX3CL1-CX3CR1 interaction mediates macrophage-mesothelial cross talk and was responsible for PD-induced peritoneal fibrosis^23^ Although hypochlorite is a fast and efficient method to induce PF; however, its molecular and cellular mechanisms were not fully understood and was considered as a “non-clinical relevant” approach to re-create PF in human patients. To address the concern and to merit the value of current model, we showed in vitro that hypochlorite-induced damages likely restricted to chemical and mechanical disruption of mesothelial cells rather than initiation of complex signal cascades for programmed death as we demonstrated in vitro that hypochlorite induced necrosis, but not apoptosis of the mesothelial cells (MeT-5A); moreover increased inflammatory cytokines IL-1β and TNF-α was measured from NaOCl-injured pigs; as NaOCl was administrated via intraperitoneal injection, a direct contact of peritoneal mesothelium with NaOCl in in vivo model likely resulted in massive necrosis as observed in in vitro assay. Moreover, overexpression of fractalkine CX3CL1 and TGFβ1 on the surface of the both parietal and visceral mesothelium observed in this study was likely due to the stimulation of IL-1β as observed in our study and was described earlier by Helmke et al.^23^. The subsequent overexpression and release of TGFβ1 from the mesothelium was evidenced from our time point immunofluorescent data and enzyme-linked immunoabsorbent assay (ELISA) analysis of peritoneal lavage. Although we did not investigate macrophage-mesothelial interactions in this study, but our data strongly supports that hypochlorite-induced PF shares similar cellular mechanism on the induction and promotion of PF as those been hypothesized in PD patients^26,29^.

This study aim to establish porcine model of PF for future evaluation of therapeutic options (e.g. cell therapy) and inhibition of PF. Based on results from this study, we considered 0.1 and 0.2% NaClO are a suitable dosage for creating PF in pig for the purpose of above-mentioned treatment assessments. Although 0.05% NaClO resulted in collagen deposition and surface thickening of liver, and caused the loss of mesothelial cells on parietal peritoneum; however, the degree of damages and pathological changes were minor and were not constantly different from control pigs. It is known that after injury, mesothelium can be partially repaired by remnant mesothelial cells via the stimulation of cell therapy^6,30,31^, therefore, sufficient number of remained mesothelial cells would affects the efficacy and the outcome of therapeutic treatments. In contrast to 0.2% NaClO, pigs injured with 0.1% NaClO exhibited consistent gross pathological changes under necropsy examination. In addition, significant increase of surface thickness and collagen deposition were measured in every organ examined. More importantly, despite apparent pathological damages were observed, around 50% of the mesothelial cells remained at the surface of both parietal and visceral peritoneum with a considerable number of αSMA^+^ myofibroblasts infiltrating at the SM compact zone. Based on above-mentioned properties, 0.1% NaClO induced a moderate to severe pathological changes in pig and is suitable for the study of cell therapy (for the repair or regeneration of mesothelium) and inhibition of fibrosis (for the reduction of αSMA^+^ myofibroblast infiltration and accumulation). Taken together, our study demonstrated for the first time that pig is useful for establishing animal model of PF at 0.1% (15 mM) concentration. Although the NaClO-induced PF model may not fully represent PD-induced PF; however, our data demonstrated high degree of similarities at both cellular and molecular mechanisms between hypochlorite and PD-induced fibrosis via the activation of IL1β-CX3CL1-TGFβ1 signal axis. Moreover, our novel porcine model provides feasibility for antemortem ultrasonographic and laparoscopic evaluation on the efficacy of potential treatments, these antemortem examinations provide instant feedback on the progression of PF and are suitable for evaluating the efficacy of treatments at any time point without sacrificing the animals. To our knowledge, these antemortem evaluations have never been performed in other animal species, and are difficult to execute in rodent species. Combine postmortem histology, marker protein assessments and quantification; detail analyses could further be performed to generate useful information about the potential and efficacy of treatments.

Despite the fact that in this study, we provided evidences supporting the cellular and molecular mechanism of hypochlorite-induced peritoneal fibrosis; however, this porcine model, like many other animal models, still can not fully represent the reality of long-term and repetitive PD fluid-induced peritoneal fibrosis in human patients. For example, chemical induced peritoneal damages and or peritonitis might be an acute injury, although we also observed similar organ fibrosis upon necropsy and histological examinations; however, the exact underlying mechanism causing fibrosis could be different from chronic stimulation by PD-fluid despite they share some degrees of similarity. Nevertheless, from our earlier publication, we showed high degrees of similarity and consistent results between 3 independent mouse models (PD-fluid induction, hypochlorite-induced and TGFβ1-induction models), therefore, we do believe that current pig model does provide and does have chance to serve as an evaluation platform for compound efficacy and therapeutic treatment evolution after mouse work and before human trial. One of the limitations of our pig model is that when compared with most of the rodent models, a relatively higher costs and experimental complexity per animal and thus low replicate numbers of the experiments will be the limitation of this model; nevertheless, higher degree of physiological and anatomical similarity of pig would certainly provide useful and valuable information to bridge the gap between human and rodent species prior to clinical trials. In conclusion, PF is a complex process involves different signaling pathways and mechanism depends on the types of stimuli, the length of exposure and the severity of the damages; unfortunately, no current animal model can represent fully the situation of human patients, therefore, when study PF, we should consider combined information from different animal models and also consider animal model which shares high physiological and anatomical similarities with human (i.e. porcine model), therefore, this study may provide an alternative evaluation platform to facilitate the understanding of human peritoneal fibrosis.

## Methods

### Chemicals, reagents, antibodies

Chemicals and reagents were obtained from Sigma Aldrich (MO, USA) unless otherwise stated. Mouse monoclonal anti-human cytokeratin (CK) clones AE1/AE3 (#M351501) was obtained from Dako/Agilent (CA, USA). Goat polyclonal anti-α smooth muscle actin (αSMA, #Ab21027), rabbit polyclonal anti-TGF-β1 (TGF-β1, #Ab92486), rabbit polyclonal anti-CX3CL1 (#Ab25088), mouse monoclonal anti- type I collagen (#Ab6308) were purchased from Abcam (Cambridge, UK). Interleukin 1β (IL1β, #201-LB) and tumor necrosis factor α (TNF-α, #PTA00) ELISA kits were obtained from R&D Systems Inc. (MN, USA). TGF-β1 ELISA kit was from BosterBio (#EK0513-PO, CA, USA). All secondary antibodies were purchased from Jackson ImmunoResearch Laboratories Inc. (PA, USA).

### Establishment of hypochlorite-induced peritoneal fibrosis model in pigs

Animal experiments were approved and carried out under the regulation and permission of IACUC protocol (NTU-106-EL-00165) at National Taiwan University (Taiwan). Thirty-two five-week-old LYD (mixed breed of Landrace-Yorkshire-Duroc) male piglets that were free from porcine epidemic diarrhea virus, porcine reproductive and respiratory syndrome virus and porcine circovirus (tested by PCR, data not shown) were purchased from conventional pig farm and housed in groups in a certified animal facility for 1 week prior to the experiments. On the day of experiment, piglets were randomly assigned to four groups (n = 5 in each experimental group, in total 20 piglets) and were weighted and sedated by intramuscular injection of xylazine (2 mg/kg) and tiletamine/zolazepam (4 mg/kg) mixture. Basic physiological parameters such as breath, heart rate, body temperature, blood oxygen level, blood pressure and electrocardiogram were monitored. To establish a NaClO (Sigma)-induced PF in pigs, NaClO was given intraperitoneally as previously described in mouse with modifications^6^. Briefly, 30 ml/kg B.W. (~ 300 ml/pig) sterilized normal saline contained 0% (v/v), 0.05% (v/v, 7.5 mM), 0.1% (v/v, 15 mM) and 0.2% (v/v, 30 mM) chemical irritant; NaClO was injected and monitored with ultrasonography (Fig. S1A, Fujifilm SonoSite, USA), post-injection evaluations were closely monitored at a daily basis (Fig. S2A).

To follow up the development and to investigate molecular mechanism of NaClO-induced PF, a time course NaClO model was also conducted based on 0.1% NaClO treatment. All pigs were housed in the animal facility as descried above and were randomly assigned to three designed time point groups (n = 3 in each experimental group including control group, 12 piglets in total). To establish base values for physiological and evaluation parameters, five days before the injection of NaClO, blood samples and abdominal lavage fluid were collected. All pigs were injected with NaClO at day 0, bloods samples and lavage fluid were collected at day 2, 4, 7 post injection. Tissue sample (i.e. parietal peritoneum, liver, duodenum) were also collected upon necropsy for further protein and histology evaluations.

### Antemortem laparoscopy evaluation

To establish antemortem evaluation to monitor the development of PF, laparoscopic examination was conducted after NaClO injection. In brief, pigs were sedated as above-mentioned. Twenty-four-gauge intravenous (iv) catheter was placed in lateral auricular vein of the ear for peri-operative administration of iv medication. The trachea was intubation and general anesthesia was maintained in pigs with 1–3% isoflurane in oxygen. The core body temperature was maintained between 36 °C to 38 °C by forced-air warming device. The vital signs, respiratory pattern, blood pressure, EKG, and end-tidal carbon dioxide (CO_2_) were monitored continuously. Normal saline was infused intravenously at a rate of 5 ml/hr/kg. Positive pressure ventilation was given during laparoscopy examination. The pigs were placed in dorsal recumbence (Fig. S2B1). After aseptical preparation, pneumoperitoneum was achieved by infusing CO_2_ via a Veress needle (Fig. S2B2). A two-port technique was adopted for laparoscopic exploration and biopsy. A 5-mm laparoscope was introduced into abdominal cavity through one of the trocars. The laparoscopic instruments, such as palpating probe and biopsy cup forceps were introduced through the other trocar (Fig. S2B3). In the exploratory, 360° endoscopic examination was performed to inspect the whole peritoneal condition. To evaluate histologically, the degree of PF, laparoscopic biopsies of liver, parietal and visceral peritoneum were collected by biopsy cup forceps. All biopsied sites were monitored for bleeding and as soon as hemostasis of biopsied site had verified, the insufflation of CO_2_ was stopped. Before cannula removal, intra-abdominal CO_2_ was purged from the peritoneal cavity. Port closure was achieved by placing 3–0 monofilament absorbable sutures (monosyn) into the muscle fascia of the body wall followed by the subcutaneous tissue. Skin incision was opposed and closed with tissue glue (Fig. S2B4).

### Pathological evaluation and immunohistochemistry staining (IHC)

After pigs were sacrificed, the severity of PF and organ adhesions was scored according to previously reported with minor modifications^28^. Modified necropsy scoring system was shown in Table 1. Tissues, including liver, intestine, omentum and parietal peritoneum, were collected and fixed in 10% neutral formalin for overnight (O/N) and processed for paraffin embedding. Ten-μm tissue sections were stained with hematoxylin and eosin (H&E) for histopathological evaluations. Pathological scoring was performed by a triple blind method by 3 certified pathologists. The severity of the lesions was graded by a 0 to 3 scale and total pathological scoring was expressed as the sum of all parameters examined. For tissue fibrosis analysis, Masson’s trichrome and collagen type I stained sections were quantitatively assessed using CellSens software (Olympus, Tokyo, Japan) and the thickness of deposited collagen was measured manually. For immunohistochemistry staining, paraffin-embedded tissue sections were used. After the deparaffinized and rehydration procedures with xylene and ethanol (100–80%), the slides will be submerged in commercially available Trilogy (Sigma, #920P-07) and heated to 121 °C for 3 min in autoclave and cool down to 45 °C for antigen retrieval. Endogenous peroxidase was removed with 3% H_2_O_2_ (Sigma, #H1009, diluted with pure methanol). After blocking by 1X normal goat serum (diluted with PBS, Jackson ImmunoResearch Laboratories Inc. PA, USA) for 1 h at room temperature (RT), antibodies against specific protein of interests was applied to the tissues for O/N incubation at 4 °C. Leica Novolink Plymer Detection System (Leica, Solms, Germany) were used to generate positive signals followed the manufacturer’s instruction and cell nuclei were counterstained with hematoxylin for evaluation.

### Indirect immunofluorescence staining and image acquisition

For indirect immunofluorescent assay (IFA), 10 μm paraffin-embedded tissue sections were processed as above-mentioned. After blocked with 1% BSA for 60 min at RT, tissue sections were permeabilized with 100% methanol at – 20 °C for 10 min. Primary antibodies incubation was carried out with O/N incubation at 4 °C. After intense washed, sections were subsequently incubated with secondary antibodies for 1.5 h at RT. Stained sections were mounted with Vectashield in the presence of diamidino-2-phenylindole (DAPI, Vector Lab, Peterborough, UK). As for negative controls, each immunoreaction was accompanied by a reaction omitting the primary antibody. All samples were evaluated with either Olympus IX83 epifluorescence microscopy or with Leica TCS SP5 II confocal scanning microscopy and analyzed with ImageJ (NIH) or CellSens software. Background subtraction was performed identically for all images (including control images) within the same set of experiments.

### Mesothelium integrity, tissue surface thickening, αSMA and type I collagen quantification

To quantify tissue surface thickening under NaClO injury, an objective multipoint measurement on the surface thickness was performed using ImageJ software as described by Su et. al^32^. For mesothelium integrity evaluation, cytokeratin (CK) was used. A mosaic tile image for the entire tissue sampled was first generated. The length (in μm) of the total tissue surface and CK^+^ region in length were measured. A ratio between CK^+^ length /total tissue surface length was calculated and expressed in percentage. To quantify the amount of myofibroblast, specific marker protein αSMA was used, total signal intensity (in pixel) was calculated and signals from the blood vessel were manually removed. The quantity αSMA^+^ signal was expressed as positive pixel/μm^2^ tissue area. For type I collagen deposition, similar pixel-based quantification method was used, the amount of type I collagen accumulation was expressed as positive pixel/μm^2^ tissue area.

### Cell culture and flow cytometry

Human mesothelial cell line, MeT-5A was obtained from ATCC (# CRL-944; Manassas, VA, USA). Cells were cultured in Medium 199 (M199, Gibco, NY, USA) supplemented with 5% fetal bovine serum, 3.3 nM epidermal growth factor (EGF) (E9644, Sigma), 400 nM hydrocortisone (H0888, Sigma), 870 nM insulin (91077C, Sigma), and 1% penicillin–streptomycin-amphotericin B (Gibco) at 37 °C in humidified atmosphere with 5% CO_2_. To investigate the effects of hypochlorite on cell fate, cellular necrosis and cell viability was assessed simultaneously with propidium iodide (PI) and annexin V as instructed (Annexin V-FITC/PI Apoptosis Detection Kit, Strong Biotech, Taipei, Taiwan). Cells were seeded in 12-well plates at a density of 3 × 10^5^ cells per well and grew for 24 h. Cells were then serum-starved for 2 h prior to hypochlorite treatments for 6–72 h. After treatments the cells were trypsinized and washed with PBS (pH 7.4). Then, incubated with 8 μl of annexin V and 2 μl of PI (final 1 μg/ml) in 100 μl binding buffer at RT for 15 min at dark. After the staining, the cells were washed, resuspended in iced-cold PBS and analyzed with FACScalibur flow cytometer (Becton and Dickinson, Pharmingen, CA, ESA). Data were further processed and analysed with the BD CellQuest Pro Software.

### Enzyme-linked immunosorbent assay (ELISA)

To examine cytokine production in the circulation (from serum samples) and local area (from abdominal lavage fluid) after NaCl-injury, target specific ELISA was performed. Both serum and abdominal lavage samples from pre- and post- NaClO injury were used for analyses, a fold change (post-/pre-) was expressed for each pair of sample, and an average of three individuals from the same treatment was calculated. For the analysis on abdominal lavage, 10 ml/kg sterile saline was infused, each pig received a gentle manual massage for 3 min after saline infusion. Infused fluid (> 50 ml) containing secretion from tissues, cytokines and cell debris were collected. After centrifuged at 1,450×*g* for 30 min at 4 °C, supernatant was collected and further concentrated with Amicon Ultra-15 3 K device (Merck, Germany) to get ~ tenfold concentrated abdominal fluid. Concentrated samples were freezed at − 80 °C for later cytokine assessment. As for the ELISA procedures, flat-bottomed Nunc Maxisorp plates were coated with 100 μl of capturing antibodies (i.e. IL-1β, TNF-α, TGF-β1) for overnight at RT, 100 µl of sample or standard were diluted in reagent diluent and added into the well containing target antibodies. After sample or standard incubations, detection antibodies were used and streptavidin-horse radish peroxidase (HRP) system was applied. Substrate buffer and stop solution were used based on manufactory instruction and optical density (O.D.) value was measured at 450 nm wavelength (EMax Plus, Molecular Devices, CA, USA).

### Statistical analyses

Data were expressed as mean ± standard deviation (S.D.). Statistical analyses were carried out using GraphPad Prism (GraphPad Software). The statistical significance was evaluated by one-way ANOVA followed with Mann–Whitney U test for 2-group comparison. And p value below 0.05 was considered as statistical different.

## Supplementary information

Supplementary file1 (PDF 18825 kb)

Supplementary file2 (MP4 57779 kb)

Supplementary file3 (MP4 9794 kb)

Supplementary file4 (MP4 6816 kb)

Supplementary file5 (MP4 9358 kb)

## Data Availability

The datasets generated during and/or analysed during the current study are available from the corresponding author on reasonable request.

## References

[1] Chang FC, Huang TM, Li WY, Lin SL (2011). Cafe-au-lait ascites in encapsulating peritoneal sclerosis. Kidney Int..

[2] Lambie MR (2016). Peritoneal inflammation precedes encapsulating peritoneal sclerosis: results from the GLOBAL Fluid Study. Nephrol. Dial. Transplant..

[3] Williams JD (2002). Morphologic changes in the peritoneal membrane of patients with renal disease. J. Am. Soc. Nephrol..

[4] Sikirica V (2011). The inpatient burden of abdominal and gynecological adhesiolysis in the US. BMC Surg..

[5] Bellingan GJ (2002). Adhesion molecule-dependent mechanisms regulate the rate of macrophage clearance during the resolution of peritoneal inflammation. J. Exp. Med..

[6] Chen YT (2014). Lineage tracing reveals distinctive fates for mesothelial cells and submesothelial fibroblasts during peritoneal injury. J. Am. Soc. Nephrol..

[7] Liao CT (2017). Peritoneal macrophage heterogeneity is associated with different peritoneal dialysis outcomes. Kidney Int..

[8] Liao CT (2016). IL-10 differentially controls the infiltration of inflammatory macrophages and antigen-presenting cells during inflammation. Eur. J. Immunol..

[9] Rosas M (2014). The transcription factor Gata6 links tissue macrophage phenotype and proliferative renewal. Science.

[10] Margetts PJ, Oh KH, Kolb M (2005). Transforming growth factor-beta: importance in long-term peritoneal membrane changes. Perit. Dial. Int..

[11] Yanez-Mo M (2003). Peritoneal dialysis and epithelial-to-mesenchymal transition of mesothelial cells. N. Engl. J. Med..

[12] Prather RS, Lorson M, Ross JW, Whyte JJ, Walters E (2013). Genetically engineered pig models for human diseases. Annu. Rev. Anim. Biosci..

[13] Kobayashi E, Hishikawa S, Teratani T, Lefor AT (2012). The pig as a model for translational research: overview of porcine animal models at Jichi Medical University. Transplant. Res..

[14] Gyongyosi M (2017). Porcine model of progressive cardiac hypertrophy and fibrosis with secondary postcapillary pulmonary hypertension. J. Transl. Med..

[15] Wang L, He FL, Liu FQ, Yue ZD, Zhao HW (2015). Establishment of a hepatic cirrhosis and portal hypertension model by hepatic arterial perfusion with 80% alcohol. World J. Gastroenterol..

[16] Seaton M, Hocking A, Gibran NS (2015). Porcine models of cutaneous wound healing. ILAR J..

[17] Dyson MC, Alloosh M, Vuchetich JP, Mokelke EA, Sturek M (2006). Components of metabolic syndrome and coronary artery disease in female Ossabaw swine fed excess atherogenic diet. Comp. Med..

[18] Noel JM (2012). Iodoacetic acid, but not sodium iodate, creates an inducible swine model of photoreceptor damage. Exp. Eye Res..

[19] Judge EP (2014). Anatomy and bronchoscopy of the porcine lung. A model for translational respiratory medicine. Am. J. Respir. Cell Mol. Biol..

[20] Kemter E, Wolf E (2015). Pigs pave a way to de novo formation of functional human kidneys. Proc. Natl. Acad. Sci. U S A.

[21] Toda N (2018). Deletion of connective tissue growth factor ameliorates peritoneal fibrosis by inhibiting angiogenesis and inflammation. Nephrol. Dial. Transplant..

[22] Oba-Yabana I (2018). Acidic organelles mediate TGF-beta1-induced cellular fibrosis via (pro)renin receptor and vacuolar ATPase trafficking in human peritoneal mesothelial cells. Sci. Rep..

[23] Helmke A (2019). CX3CL1-CX3CR1 interaction mediates macrophage-mesothelial cross talk and promotes peritoneal fibrosis. Kidney Int..

[24] Wang L (2016). Inhibition of EGF receptor blocks the development and progression of peritoneal fibrosis. J. Am. Soc. Nephrol..

[25] Wang L, Zhuang S (2015). The role of tyrosine kinase receptors in peritoneal fibrosis. Perit. Dial. Int..

[26] Zhou Q, Bajo MA, Del Peso G, Yu X, Selgas R (2016). Preventing peritoneal membrane fibrosis in peritoneal dialysis patients. Kidney Int..

[27] Levine S, Saltzman A (1996). Abdominal cocoon: an animal model for a complication of peritoneal dialysis. Perit. Dial. Int..

[28] Fang CC (2012). Fibrin-Induced epithelial-to-mesenchymal transition of peritoneal mesothelial cells as a mechanism of peritoneal fibrosis: effects of pentoxifylline. PLoS ONE.

[29] Fielding CA (2014). Interleukin-6 signaling drives fibrosis in unresolved inflammation. Immunity.

[30] Mutsaers SE, Prele CM, Pengelly S, Herrick SE (2016). Mesothelial cells and peritoneal homeostasis. Fertil. Steril..

[31] Lua I, Li Y, Pappoe LS, Asahina K (2015). Myofibroblastic conversion and regeneration of mesothelial cells in peritoneal and liver fibrosis. Am. J. Pathol..

[32] Su X (2014). The PPARbeta/delta agonist GW501516 attenuates peritonitis in peritoneal fibrosis via inhibition of TAK1-NFkappaB pathway in rats. Inflammation.

