# Supplementary material for: Hypochlorite-induced porcine model of peritoneal fibrosis through the activation of IL1β-CX3CL1-TGFβ1 signal axis
Source: Sci Rep. 2020 Jul 13;10:11496. doi: 10.1038/s41598-020-68495-0 (PMC7359301; doi:10.1038/s41598-020-68495-0)

Hypochlorite-induced Porcine Model of Peritoneal Fibrosis Through The Activation of IL1β-CX3CL1-TGFβ1 Signal Axis

Yu-Ting Hsu[1,2,#], Ching-Ho Wu[1,3,#], Chun-Yuan Chao[1,2,#], Yu-Syuan Wei[1,2], Yen-Chen Chang[1,4], Yi-Ting Chen[5,6], Shuei-Liong Lin[5,6,7,9], Su-Yi Tsai[8,9], Ya-Jane Lee[1,3], Pei-Shiue Tsai[1,2,9,*]

Running title: Porcine model of hypochlorite-induced peritoneal fibrosis

[1]Department of Veterinary Medicine, School of Veterinary Medicine, National Taiwan University, 10617 Taipei, Taiwan

[2]Graduate Institute of Veterinary Medicine, School of Veterinary Medicine, National Taiwan University, 10617 Taipei, Taiwan

[3]Graduate Institute of Veterinary Clinical Science, School of Veterinary Medicine, National Taiwan University, 10617 Taipei, Taiwan

[4]Graduate Institute of Molecular and Comparative Pathobiology, School of Veterinary Medicine, National Taiwan University, 10617 Taipei, Taiwan

[5]Department of Internal Medicine, National Taiwan University Hospital, 10002, Taipei, Taiwan

[6]Department of Integrated Diagnostics & Therapeutics, National Taiwan University Hospital, 10002, Taipei, Taiwan

[7]Graduate Institute of Physiology, College of Medicine, National Taiwan University, 10051 Taipei, Taiwan

[8]Department of Life Science, College of Life Science, National Taiwan University, 10617 Taipei, Taiwan

[9]Research Center for Developmental Biology and Regenerative Medicine, National Taiwan University, 10617 Taipei, Taiwan

[#]Authors contributed equally

[*]**Corresponding author**:

Pei-Shiue Jason Tsai

Department of Veterinary Medicine, Graduate Institute of Veterinary Medicine, School of Veterinary Medicine, National Taiwan University, 10617 Taipei, Taiwan

Email: psjasontsai@ntu.edu.tw, Tel: 886(0)2 33661806; Fax: 886(0)2 23661475

ORCID: 0000-0001-8217-6285

Supplementary Figure 1 Operation of NaClO-injection and pathological changes of parietal peritoneum after NaClO-injury.

A. Ultrasonography was used to monitor the injection of sodium hypochlorite. Pre-injection evaluation was carried out thoroughly for the comparison with post-injection analysis. Injected sodium hypochlorite was monitored and marked with asterisks. I: intestine; L: liver. **(B)** 3D reconstruction images confirmed the integrity of mesothelium and accumulation of αSMA^+ myofibroblasts in the submesothelial tissue of control and hypochlorite-injured pigs.

Supplementary Figure 2 Laparoscopy procedures for antemortem evaluation of peritoneal fibrosis.

A. (1-2) Sodium hypochlorite was diluted with sterilized saline and was administrated via intraperitoneal injection in anesthetized pigs. (3) Injection site was indicated with arrow. (4) Pigs were placed in a high stand recovery room with heated lamp after NaClO injection. **B.** (1) Antemortem laparoscopy evaluation of peritoneal fibrosis was carried out as described in methods and material under aspectic condition. Incision site was marked with arrow. (2) Pneumoperitoneum was achieved by infusing CO_2 via a Veress needle. (3) A two-port technique was adopted for laparoscopic exploration and biopsy. A 5-mm laparoscope was introduced into abdominal cavity through one of the trocars. The laparoscopic instruments, such as palpating probe and biopsy cup forceps were introduced through the other trocar. (4) Discontinued suture was performed after thorough antemortem laparoscopy evaluation.

Supplementary Video 1 Laparoscopy examination of peritoneal cavity of control pig (1 week after normal saline injection). Representative video was presented.

Supplementary Video 2 Laparoscopy examination of peritoneal cavity of 0.05% NaClO-injured pig (1 week after injection). Representative video was presented.

Supplementary Video 3 Laparoscopy examination of peritoneal cavity of 0.1% NaClO-injured pig (1 week after injection). Representative video was presented.

Supplementary Video 4 Laparoscopy examination of peritoneal cavity of 0.2% NaClO-injured pig (1 week after injection). Representative video was presented.

Supplementary Figure 1

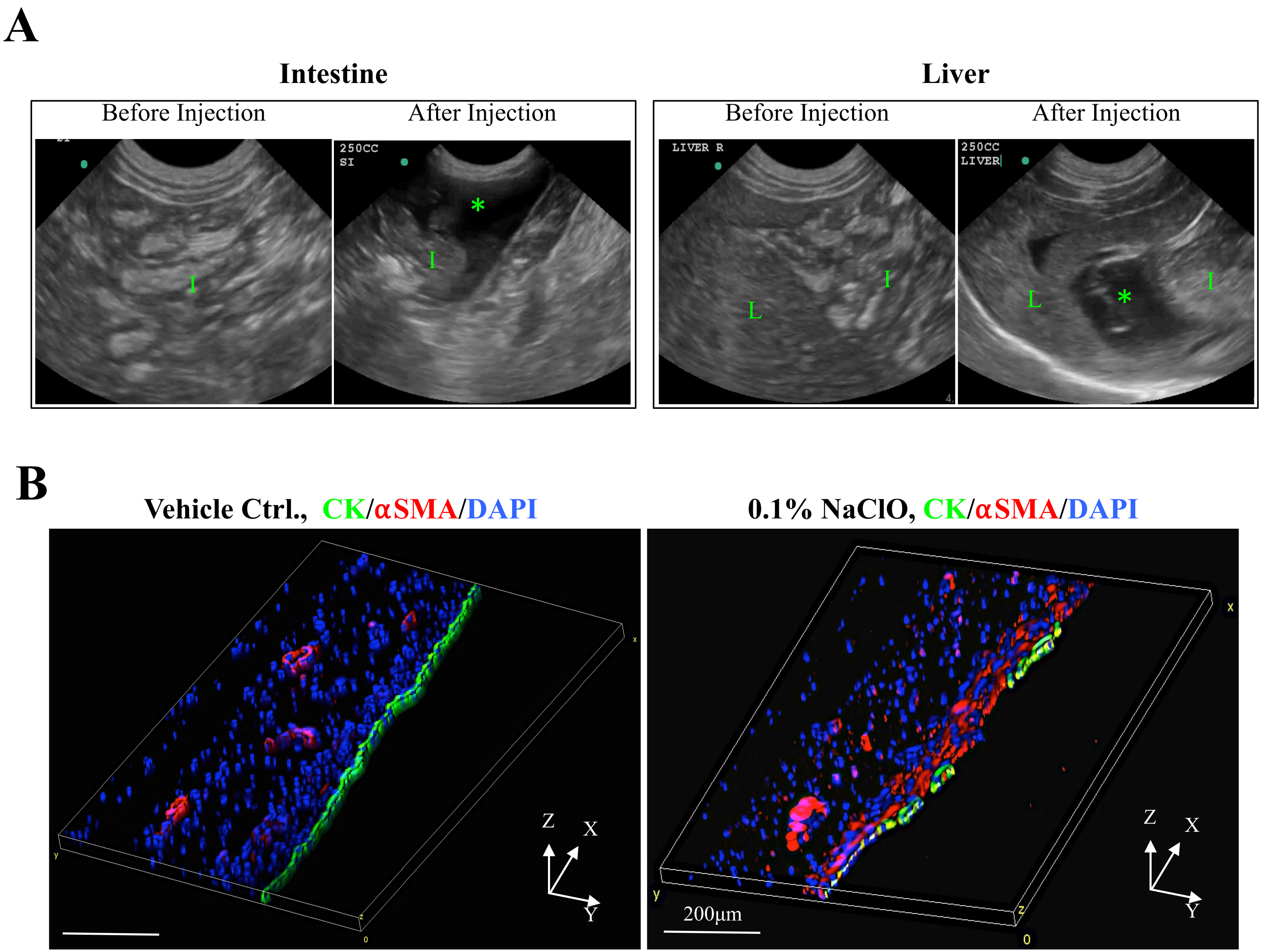

Supplementary Figure 2

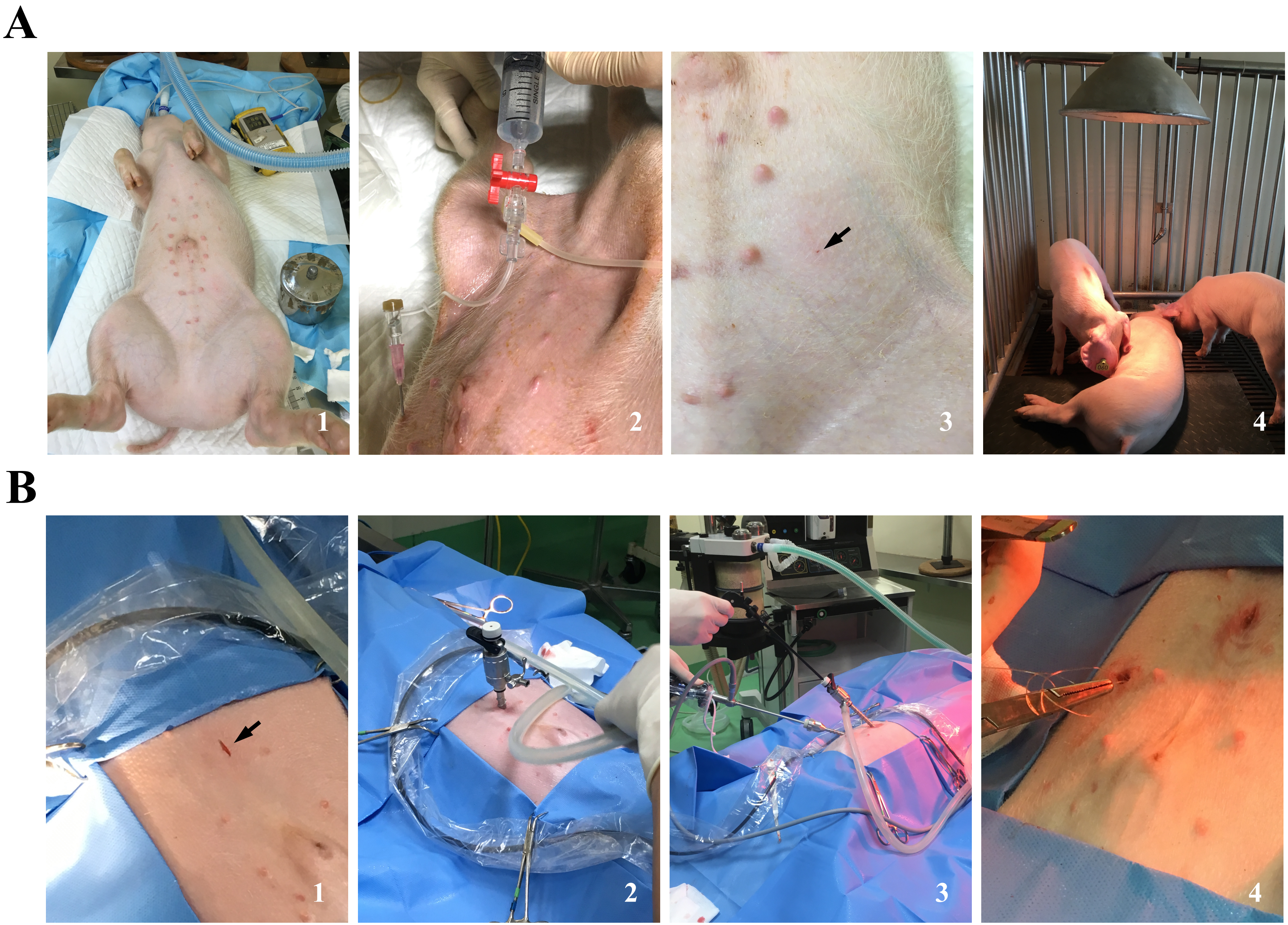

Supplement: Supplementary file 1 — Supplementary file1 (PDF 18825 kb) [file 41598_2020_68495_MOESM1_ESM.pdf]
